# Virtual Wireless Sensor Networks: Adaptive Brain-Inspired Configuration for Internet of Things Applications

**DOI:** 10.3390/s16081323

**Published:** 2016-08-19

**Authors:** Shinya Toyonaga, Daichi Kominami, Masayuki Murata

**Affiliations:** 1 Graduate School of Information Science and Technology, Osaka University, 1-5 Yamadaoka, Suita, Osaka 565-0871, Japan; murata@ist.osaka-u.ac.jp; 2 Graduate School of Economics, Osaka University, Osaka 560-0043, Japan; d-kominami@econ.osaka-u.ac.jp

**Keywords:** virtual topology, human-brain network, routing, hierarchical modular structure, small-world properties

## Abstract

Many researchers are devoting attention to the so-called “Internet of Things” (IoT), and wireless sensor networks (WSNs) are regarded as a critical technology for realizing the communication infrastructure of the future, including the IoT. Against this background, virtualization is a crucial technique for the integration of multiple WSNs. Designing virtualized WSNs for actual environments will require further detailed studies. Within the IoT environment, physical networks can undergo dynamic change, and so, many problems exist that could prevent applications from running without interruption when using the existing approaches. In this paper, we show an overall architecture that is suitable for constructing and running virtual wireless sensor network (VWSN) services within a VWSN topology. Our approach provides users with a reliable VWSN network by assigning redundant resources according to each user’s demand and providing a recovery method to incorporate environmental changes. We tested this approach by simulation experiment, with the results showing that the VWSN network is reliable in many cases, although physical deployment of sensor nodes and the modular structure of the VWSN will be quite important to the stability of services within the VWSN topology.

## 1. Introduction

For the future Internet of Things (IoT) environment, the effective integration of various types of networks will be an important consideration. In addition to the co-existence of wired and wireless links, domains may involve sensor nodes and actuator nodes from multiple vendors. Moreover, applications running over such mixed networks will make different demands of the network, such as locational information or other node-specific information.

To realize the IoT environment, wireless sensor networks (WSNs) will be a crucial technology to allow collecting and acting on environmental information. WSNs will be integrated into future communication infrastructure [1]. Here, however, networks are considered to be constructed independently to provide service within a local area.

For such heterogeneous WSNs to be consolidated into infrastructure and to share physical sensor substrates across multiple IoT applications, virtualization of WSNs is one solution. In virtualization methods, the functionality of a WSN is split into two parts: the physical infrastructure of sensors and the applications, which rely on the aggregated resources. The main advantage of virtualizing WSNs is that this provides the ability to realize shared infrastructure that can satisfy various service demands [2,3].

For example, sensor substrates deployed for fire-detection or fire-tracking applications can be shared between homeowners and city administration [4]. In extant task-oriented WSNs, redundant sensor nodes are deployed in the shared domain, and the duplicated nodes are chosen according to application. This is done because the required granularity or service demands of the distinct users are different, but such redundant deployment is inefficient. Virtualization can eliminate tight coupling between applications and resources, which enables multi-application use of existing sensor substrates. Moreover, virtualization of WSNs provides a new business model, sensor-as-a-service, for both infrastructure owners and service providers [5].

Virtualization of WSNs has been studied by many researchers. The Federated Secure Sensor Network Laboratory (FRESnel) [6] and Virtualized dIstributed plaTfoRms of smart Objects (VITRO) [7] projects, in particular, are focused on the virtualization of WSNs. FRESnel aims to federate large-scale WSNs and enable the simultaneous running of multiple applications. VITRO aims to provide dynamic cooperation among sensor nodes by dividing WSNs into physical sensor substrates and applications.

In previous work on the virtualization of WSNs, virtualization at two levels has been studied: node and network [4]. Node-level virtualization methods enable a single node to concurrently process multiple applications. Approaches to node-level virtualization can be divided into three classes according to the element that provides concurrency: a virtual machine [8], the operating system [9] or middleware [10]. Under network-level virtualization, the virtual network for running a single application consists of a subset of sensor nodes. Network-level virtualization results in efficient use of resources because the nodes not used by one application can be used by applications in other virtual networks. Approaches to network-level virtualization can be divided into two classes: overlay-based solutions [11] and cluster-based solutions [12].

Within this system of classification, we aim for overlay-based network-level virtualization. Many other researchers have proposed overlay-based approaches to network-level virtualization [3,11]. Those approaches focus mainly on providing a framework that allows the sharing of physical sensor resources. Although improved manageability has been mentioned as a reason for virtualizing networks, the method of constructing a virtual wireless sensor network (VWSN) topology for each application in an overlay layer has not been discussed enough. Moreover, within the IoT environment, changes to traffic patterns, variation in traffic demand and the addition or removal of virtual nodes can all occur. Because of this, providing stable applications remains difficult when using the currently-existing approaches, in which only required resources (i.e., without redundancy) are assigned to a user. Reliability, even in the face of such environmental changes, is important. For that reason, we focus on a means of constructing a robust and adaptive VWSN topology for applications.

To ensure robust connectivity, each node should know all routes to the other nodes. However, keeping this information up to date would require all nodes to exchange or maintain their information with each other, resulting in heavy traffic. Especially for energy-scarce WSNs, strategies with low overhead are required. Therefore, we need to construct reliable VWSNs while keeping the overhead low for nodes. From the viewpoint of VWSN managers, the efficient distribution of sensor substrates is necessary in order to maximize per-sensor benefit. Therefore, a desirable VWSN construction method will be able to form as many virtual networks from the finite sensor substrates as possible and allow many users and applications to share the physical network. One solution to this type of problem is to use a hierarchical structure. Such structures offer both low overhead and high manageability because they can abstract the physical network and allow nodes to use their information in multiple applications.

In our previous work, we proposed a method of construction of a VWSN topology that offers robust connectivity against node removal [13]. Our approach is inspired by brain network features, and we focus particularly on the similarity between the hierarchical modular structure of brains and the modular structure that emerges from the integration of local networks.

A brain network has many structural properties, such as heavy-tailed degree distribution, rich clubs, clustering, small-world properties, hierarchical modular structure, and so on [14]. Especially, small-world properties and hierarchical modular structure contribute to the evolvability of the brain network and high communication efficiency in the global area with low metabolic cost [15]. Our conclusive goal is autonomously evolving VWSN according to the environmental changes. When congestion or event-driven burst traffic occurs or the physical topology changes according to the addition/removal/move of nodes, the resources for the virtual topology, such as time slots for the application, the number of physical paths assigned to a virtual link or new virtual links for new physical topology, should be reassigned to meet the environment. As the first step toward this goal, we introduce the structural properties of the brain network into a VWSN topology.

Our approach consists of two steps: constructing modules with small-world properties and integrating the modules hierarchically. Our idea is a bottom-up design of the hierarchical structure, which is inspired by the brain. The modules in the brain network consist of sub-modules, which have a close correlation in terms of their function. In our proposed structure, a higher-tier module consists of sub-modules that are assigned to sub-applications, and the module describes an integrated system. We expect that such a modular structure taking into account modules’ functions contribute to resource-efficient solutions for users’ demands. However, the evaluation of resource efficiency is out of scope here. We evaluate the topology constructed by our proposal without an autonomous configuration because the performance characteristics of the constructed VWSN topology itself are primarily important.

We investigate connection patterns within each module and between modules. We showed that the VWSN topology constructed by our proposal is robust against node removal on both connectivity and path length in our previous work [13].

In this paper, we show an overall architecture for constructing and running VWSN services on a VWSN topology as a further investigation of our proposed method. Our main focus is providing a user a reliable VWSN network, and we show one solution providing a user IoT resources by dividing providers into infrastructural and VWSN providers. Then, we show how we connect modules to construct a reliable VWSN network. Our contributions are as follows.

- Our main idea for constructing a robust VWSN topology is shown in our previous work [13]. In this paper, we show a series of procedures for running services on the VWSN topology and demonstrate the feasibility of our proposal by simulation, including from user-level requests for a new virtualized WSN to packet-level behaviors.
- We evaluate the features of our proposed method, especially adaptivity. Here, we define the adaptivity of the constructed VWSN as the ability to quickly recover its function, where various types of traffic exist. Supposing a node failure scenario, we show that our proposal can be adaptive by proactively setting up routers and reactively recovering them. The hierarchical modular structure of VWSNs allows a memory-efficient routing method with low overhead by restricting the flooding area used during route recovery. We also show that adaptivity can be low even when the VWSN network has robust connectivity.

Note that our focus is not the proposal of a routing or route recovery method itself. The routing method and the route recovery method shown in this paper are used for clarifying the structural problem of our VWSN network that harms the reliability of the VWSN topology. Therefore, another routing method can do. Moreover, we assume that each application can use different routing strategies, and other protocols can also be configured by users according to their demands. Clearly, it should be in the virtualization scenario.

The rest of this paper is organized as follows. Related work is shown in Section 2. In Section 3, we present a use-case scenario and give an overview of the virtualization of WSNs, which is the focus of this paper. The description of the method that we propose for constructing a VWSN topology inspired by brain networks is shown in Section 4. In Section 5, we show a routing algorithm that combines a hierarchical modular structure with a simple method for the discovery of an alternative route after node failure. We evaluate our proposal against the use-case scenario of Section 6. In Section 7, we conclude this paper and describe future work.

## 2. Related Work

Many researchers have worked on the virtualization of wired networks, such as the local area network (LAN) or data center. The virtual local area network (VLAN) or virtual private network (VPN) are well-known examples of the virtualization for wired networks [16]. The overlay network that has a virtual topology constructed on the physical topology is also this kind of example [17]. Constructing an overlay network can be seen as a virtual network embedding problem, which consists of virtual node mapping and virtual link mapping. Virtual nodes are allocated in physical nodes in virtual node mapping, and virtual links connecting virtual nodes are allocated in physical paths connecting the corresponding physical nodes in the virtual link mapping. In virtual network embedding, the request of constructing an overlay network is described as a graph with the required resources of virtual nodes and virtual links, such as CPU or bandwidth. In the wireless scenario, however, resource abstraction of links, such as bandwidth, is challenging because of unstable channel quality [16]. Interference and fading of wireless signals are inevitable without sophisticated time synchronization of nodes and can degrade the performance of virtual networks. Moreover, in the future IoT environment, there are various applications, and burst, periodic and time-varying traffic coexist. Therefore, it is not realistic to reserve the finite size of resources in the environment, and the existing methods for wired networks are not applicable straightforwardly.

In the topology control of a WSN, tree-based and clustering tree-based approaches are proposed for energy-efficient data collection and node-to-node routing [18]. In such a cluster-based topology, however, packets for inter-cluster communication need to go through cluster heads, which can be a single failure. Jameii et al. applied a multi-objective optimization scheme to the topology control of a WSN [19]. Their focus is to provide a set of Pareto-optimal solutions that optimize four competitive objectives, the number of active nodes, coverage, connectivity and energy conservation, by adjusting the communication ranges of sensor nodes and sleep scheduling of sensor nodes. In addition to the multi-objective optimization algorithm, they introduce a learning automata for the dynamical configuration of the topology according to the environmental changes. Although the simulation evaluation showed the best performance compared to the existing approaches, the discussion about routing overhead and computational overhead is lacking.

For decoupling IoT networks into the control plane and the data plane, software-defined networking (SDN) technologies are discussed. Qin et al. proposed an SDN controller for managing heterogeneous IoT networks [20]. They define a resource matching strategy between abstract task description and resource specifications and flow scheduling by using a heuristic algorithm until the algorithm finds a feasible solution for the request. Because the controller estimates the end-to-end flow performance before resource allocations, their proposal shows better throughput, delay and jitter than existing ones. Jararweh et al. proposed a software-defined IoT framework, which exploits several software defined systems, such as SDN, software-defined storage and software-defined security [21]. Their focus is to simplify the IoT management and to solve problems involved in the big data scenario. However, specific evaluations of the model were not examined. Other virtualization schemes for eliminating the tight-coupling of applications and physical substrates in IoT networks are also proposed. However, to our knowledge, how to construct the VWSN topology that supports reliable communication has not been discussed enough.

Therefore, we provide a new viewpoint to make VWSN topology reliable by introducing a brain-inspired structure, and we conduct simulation evaluation with considering environmental changes and packet-level behavior, including control-plane and data-plane packets. We also discuss the feasibility and overheads of our proposed architecture. In this paper, however, we do not consider how to get resource efficiency or energy efficiency, which will be more critical in the virtualization scenario. Although we discuss approaching our considering world from the current real world in Section 6.7, how to solve these issues is out of our scope, but should be future work.

## 3. Scenario for Providing a VWSN

In this paper, we adopt the concept of the virtualization of WSNs as shown in [2] and assume that the virtualization of WSNs is realized by two types of providers. One type is an infrastructure provider that manages physical sensor nodes and physical WSNs; the other is a VWSN provider that constructs a VWSN by aggregating physical substrates from multiple infrastructure providers. We assume that a VWSN provider is responsible for constructing a VWSN topology for an application.

**Figure 1:**
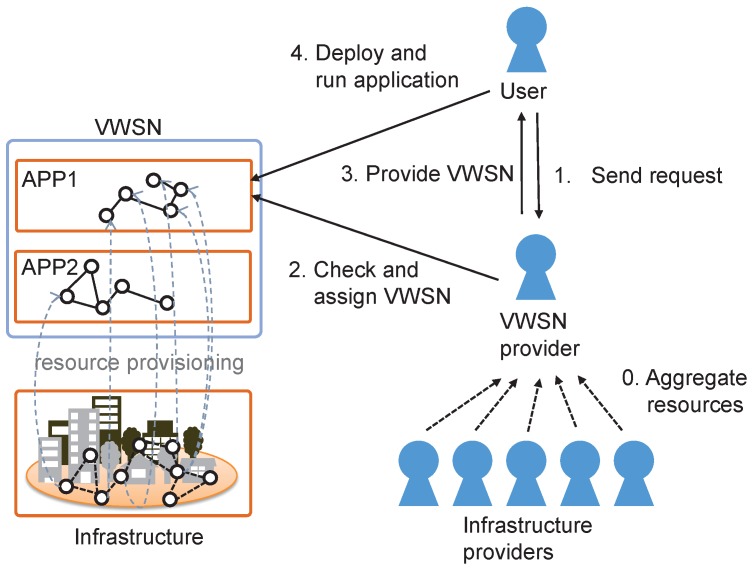
Providing a virtual wireless sensor network (VWSN) to a user.

In this paper, a VWSN is provided to handle user requests as shown in Figure 1. A user who wants to develop a new application selects a set of source nodes and destination nodes. This set of nodes can be abstracted through an interface provided by the VWSN provider. Then, the user sends a request to construct a new VWSN, and this message is received by a VWSN provider. When the user requests abstract resources, such as the periodic report of humidity and temperature in the northeast area of Tokyo in Japan, the VWSN provider selects a set of source and destination nodes corresponding to the user’s request. The request message contains the traffic pattern between the source nodes and destination nodes, as well as the required degree of reliability. The traffic patterns describe the way that data packets are generated, such as periodic or event-driven scheduling. When the VWSN provider receives the request, it checks whether there are enough resources to construct a VWSN that satisfies the request. When there are not enough resources, the VWSN provider rejects the request. Otherwise, the VWSN provider constructs a topology and assigns at least one physical path to a virtual link. When high reliability is demanded, redundant physical paths can be assigned to a virtual link. When a low number of redundant physical paths is assigned to a virtual link, the resources of sensor nodes are more fully utilized, but low redundancy may lower reliability. At this time, the VWSN provider determines which nodes the user will be allowed to use. The allowed ones include source nodes, destination nodes and relay nodes for communication between source and destination nodes. Once the set is decided, the VWSN provider calculates routing tables for each node in the constructed VWSN and sends the information, including the traffic pattern, to the gateway that connects the Internet and WSNs and may translate protocols to communicate with nodes in WSNs. The gateway sends the information to each node in the VWSN. Over time, each node in the VWSN acts as a resource for the VWSN and sends data according to the received traffic pattern.

As described in Section 2, it is true that it is hard to manage resources completely because of unstable channel quality and the time-varying traffic pattern. One solution for determining whether the user’s request can be met or not is to limit the number of applications running on each module or node.

## 4. A Method for Configuring VWSNs to Use the Properties of Brain Networks

In this section, we show the method for constructing a VWSN topology by using the structural properties of human-brain networks. First, we describe some topological properties possessed by human-brain networks and explain the expected advantages of introducing them into WSNs in Section 4.1. Then, the brief explanation of the VWSN topology construction method proposed in our previous work [13] is shown in Section 4.2.

### 4.1. Human-Brain Networks

A brain network possesses a modular community structure and has small-world properties. The modular community structure offers robustness, adaptivity and evolvability. The small-world properties offer high communication efficiency in both local and global domains, as well as local robustness [15,22,23,24,25,26].

#### 4.1.1. Modular Community Structure

A brain network is spatial, which means that the length of a connection is limited by the metabolic costs associated with establishing and maintaining the connection [14,22,27]. As a consequence, many short-distance links are constructed and maintained in preference to long-distance links. Within the modular community structure possessed by brain networks, connections are dense and short within modules, but sparse and long between modules. Further, this type of structure is hierarchical [15,22].

The modular structure in human-brain networks offers various advantages. The high density of connections within each module ensures robust connectivity, including many alternative routes between pairs of nodes within the same module. Moreover, connection distance and communication delay can be adjusted quickly by configuring long-distance inter-module links [22]. When cognitive demand increases, costly long-distance links are constructed so as to acquire a more efficient structure; when demand decreases, the structure becomes more highly clustered and, hence, less costly. This feature contributes most especially to evolvability.

The introduction of a hierarchical modular structure to WSNs is expected to result in a topology that can evolve adaptively to changes in resources and traffic demand. Because WSNs, like brain networks, are spatial networks, sensor nodes deployed in a close area will be densely interconnected when a hierarchical modular structure is used. Therefore, the topology of a WSN generally possesses many alternative routes, resulting in robust connectivity. Moreover, the hierarchical structure is suitable for the horizontal integration of virtual networks, which increases the reusability of virtual resources. To integrate multiple VWSNs into one VWSN, a new tier can be overlaid onto the existing VWSNs, which are then connected in the added tier. In the application that uses this integrated VWSN, each node can reuse most of the routing tables assigned by the component VWSNs.

#### 4.1.2. Small-World Properties

Small-world networks are characterized by properties, such as short average path length and a high clustering coefficient. The average path length (APL) is defined as

(1) $$\mathrm{APL}=\frac{1}{N(N-1)}\sum_{i,j}sd\left(i,j\right),$$

where *N* is the number of nodes and sd(i,j) is the lowest hop count between node *i* and node *j*. A low APL indicates highly efficient global communication. The clustering coefficient (CC) is defined as

(2) $$\mathrm{CC}=\frac{1}{N}\sum_{i}\frac{2e_{i}}{k_{i}\left(k_{i}-1\right)},$$

where $e_{i}$ denotes the number of links between neighbors of node *i*, and $k_{i}$ is the degree of node *i*. The degree of a node is the number of neighboring nodes connected with the node by wireless or wired links. A high CC indicates that nodes in local regions are densely connected, which contributes to local communication efficiency.

In brain networks, densely-connected nodes within a module contribute to a high CC, which leads to efficient, segregated information processing and synchronization. Myelinated long-distance links with high electric conductivity (i.e., high reliability, high speed, long-distance links) are one contributor to global communication efficiency in brain networks [22]. The short communication delay of this type of link enables close cooperation between different regions.

To model this property in a VWSN topology, we introduce a virtual long-distance connection that connects two physically-distant sensor nodes. This leads to a reduction in communication delays across the whole network because it reduces the APL while many parts of the network keep their overheads low.

### 4.2. VWSN Topology Construction Method

**Figure 2:**
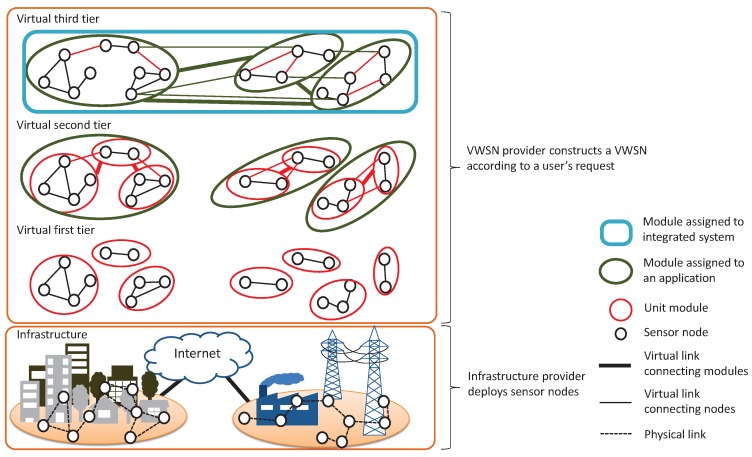
Example of a hierarchical VWSN topology.

As discussed above, a topology with the small-world properties will have high global communication efficiency, and a highly modular topology will have efficient segregated information processing and robust connectivity. We previously proposed a means of constructing a VWSN topology with small-world properties and high modularity [13]. Here, we integrate modules hierarchically to construct a VWSN topology. At the same time, we add long-distance links to a clustered topology to give the VWSN topology the small-world properties at each tier. Figure 2 shows an example of hierarchical VWSN construction. The first tier of the VWSN is a network in the minimum-unit module. The second-tier VWSN is constructed by integrating unit modules. The third-tier VWSN is constructed by integrating virtual sensor networks deployed for sub-applications.

In this section, we briefly explain the method proposed in [13]. We assume that adding multi-hop wireless or wired links contributes to reachability between any two nodes. After this, a virtual link is mapped to the shortest physical path in an infrastructural topology. Note that we consider that the physical topology of WSN is the unit disk model and does not have small-world properties. We construct a virtual network having the small-world properties that contribute to a short average path length by embedding the small number of long-distance virtual links.

First, a VWSN provider defines a set of nodes for a user application. The constructed VWSN must contain a set of source and destination nodes requested by the user, a set of relay nodes for communication between source and destination nodes and redundant nodes according to the user’s demand for reliability. When these sets of nodes have been determined, the VWSN provider selects a set of modules to construct a VWSN and integrates them by embedding virtual links between them.

Construction of an *N*-th-tier VWSN topology can be divided into two smaller subproblems. In the first problem, we regard each (N−1)-th-tier VWSN as one module ($M_{i}^{N-1}$) in the *N*-th tier, where *i* indicates the specific module, and the choice to be made is how to connect pairs of modules. In the second problem, sensor nodes are to be mapped to the endpoints of *N*-th-tier virtual links. In this, $M_{*}^{0}$ denotes a sensor node and $M_{*}^{1}$ denotes a module clustered by applying the Newman algorithm [28].

For the first problem, we construct an *N*-th-tier virtual topology by adding virtual links between (N−1)-th-tier modules. First, an initial virtual topology is constructed: when two nodes belonging to different (N−1)-th-tier modules are connected by a physical link, an (N−1)-th-tier virtual link is embedded between these (N−1)-th-tier modules. Second, new (N−1)-th-tier virtual links are embedded into the initial virtual topology according to a preferential attachment rule. The probability of adding an (N−1)-th-tier virtual link between $M_{i}^{N-1}$ and $M_{j}^{N-1}$ is

(3) $$p_{\mathrm{intra}}^{N}\left(M_{i}^{N-1},M_{j}^{N-1}\right)=\frac{\frac{G^{intra}\left(k_{M_{i}^{N-1}},k_{M_{j}^{N-1}}\right)}{F(h_{M_{i}^{N-1},M_{j}^{N-1}})}}{\sum_{e_{M_{a}^{N-1},M_{b}^{N-1}}\in {\bar{E}}^{N}}\frac{G^{intra}\left(k_{M_{a}^{N-1}},k_{M_{b}^{N-1}}\right)}{F(h_{M_{a}^{N-1},M_{b}^{N-1}})}}.$$

Here, (N−1)-th-tier modules $M_{i}^{N-1}$ and $M_{j}^{N-1}$ belong to the same *N*-th-tier module, ${\bar{E}}^{N}$ is the set of virtual links in the graph complement of the *N*-th-tier initial virtual topology and *F* is a cost function $F\left(d\right)=e^{d/d_{x}}$, where $d_{x}$ is a constant parameter characterizing distance constraints. $h_{M_{i}^{N-1},M_{j}^{N-1}}$ denotes the minimum hop count between $M_{i}^{N-1}$ and $M_{j}^{N-1}$ in the *N*-th-tier initial virtual topology, and $k_{M_{i}^{N-1}}$ denotes the degree of $M_{i}^{N-1}$. The degree of an *N*-th-tier module is the number of neighboring *N*-th-tier modules connected with the module by *N*-th-tier virtual links. Strategy function $G^{intra}$ is a function for embedding a new link preferentially according to the degrees of the endpoint modules. The strategy intra for adding a new link can be any of “high-high (hh)”, “low-low (ll)” and “high-low (hl)”. When intra=hh, a pair of higher degree modules is selected preferentially for a new link, and when intra=ll, a pair of lower degree modules is selected for a new link. When intra=hl, a higher degree module and another lower degree module are selected preferentially and connected. The definitions of $G^{intra}$ for each possibility are as follows:

$$\begin{array}{ccc} G^{hh}\left(k_{i},k_{j}\right) & = & k_{i}\cdot k_{j}\\ G^{ll}\left(k_{i},k_{j}\right) & = & k_{i}^{-1}\cdot k_{j}^{-1}\\ G^{hl}\left(k_{i},k_{j}\right) & = & \max\left(k_{i},k_{j}\right)\cdot \left|k_{i}-k_{j}\right| \end{array}$$

The number of added *N*-th-tier virtual links is $\left\lceil C_{\mathrm{intra}}^{N}|E_{0}^{N}|\right\rceil$, where $|E_{0}^{N}|$ is the number of links embedded in the *N*-th-tier initial virtual topology and $C_{\mathrm{intra}}^{N}$ is a constant satisfying $0<C_{\mathrm{intra}}^{N}\leq 1$.

For the second problem, we describe a method for mapping the endpoints of an *N*-th-tier virtual link to sensor nodes. In this method, we recursively select the endpoints of an *N*-th-tier virtual link from its submodules until the endpoint nodes are determined. We define the probability $p_{\mathrm{inter}}^{N}\left(M_{i}^{N-1},M_{j}^{N-1}\right)$ of mapping an *N*-th-tier virtual link to an (N−1)-th-tier virtual link between $M_{i}^{N-1}$ and $M_{j}^{N-1}$ in the same way as Equation (3). The strategy inter for mapping can be one of “High-High (HH)”, “Low-Low (LL)” and “High-Low (HL)”, with the same meanings as “hh”, “ll” and “hl”, respectively, but applied to links between modules. The number of virtual links for each mapping is $\left\lceil C_{\mathrm{inter}}^{N}\left(E^{M_{x}^{N}}+E^{M_{y}^{N}}\right)\right\rceil$, where $E^{M_{x}^{N}}$ denotes the number of (N−1)-th-tier virtual links in the *N*-th-tier VWSN topology of $M_{x}^{N}$, and $C_{\mathrm{inter}}^{N}$ is a constant value satisfying $0<C_{\mathrm{inter}}^{N}\leq 1$. We call an endpoint node of a virtual inter-module link a connected node.

Next, we assign a virtual link to one of physical shortest paths, and the routing tables are constructed by the method mentioned in the following section. From here, we suppose that any node can be reassigned to a relay node of a physical path of any virtual link in the event of node failure. Therefore, we assume that the user requires the maximum degree of reliability. The route recovery method is also described in the following section.

## 5. Routing over the Hierarchical Virtual Topology

In a routing protocol for a hierarchical topology, a within-module network can be aggregated into an abstract topology. Owing to this abstraction of modules, it is not necessary for each sensor node to hold information about every path to each node. This improves the efficiency of memory usage. To take advantage of this, we use minimum-weight path routing, considering the modular structure of a VWSN topology.

In Section 5.1, we describe an overview of the routing algorithm we use. In Section 5.2, we show the routing tables that each node needs to hold for deciding a forwarding node, and in Section 5.3, we show a path recovery method. We show the details of the packet format and the definition of path weight used in our simulation evaluations in Appendix A and Appendix B, respectively.

### 5.1. Overview

**Figure 3:**
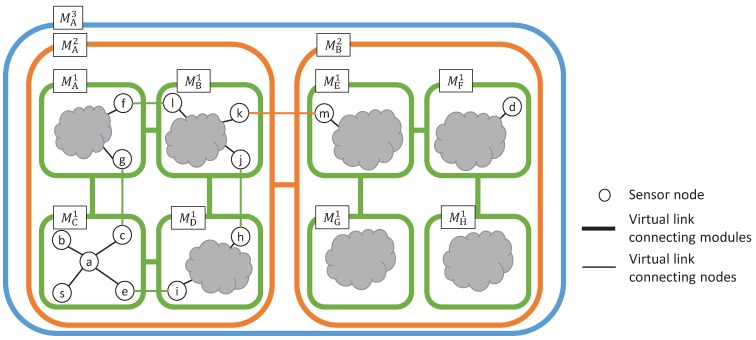
Example of a hierarchical topology.

In this section, we give an overview of our routing algorithm. Figure 3 shows an example of a three-tiered virtual topology. Each node belongs to one module in each tier. For example, node *a* belongs to module $M_{C}^{1}$ in the first tier, module $M_{A}^{2}$ in the second tier and module $M_{A}^{3}$ in the third tier. The end nodes of each higher-tier link are assigned to physical nodes. In Figure 3, the end nodes of the first-tier link ($M_{A}^{1}$,$M_{C}^{1}$) are assigned to nodes *g* and *c*, and the end nodes of the second-tier virtual link ($M_{A}^{2}$,$M_{B}^{2}$) are assigned to nodes *k* and *m*. In this paper, as mentioned in Section 3, the VWSN provider calculates the routing tables and sends them along with the traffic patterns to each node. At this time, the VWSN provider informs source nodes of which modules the corresponding destination node belongs to in each tier. Then, source nodes can refer to the information when sending a data packet to their destination nodes.

Below, we show a method for choosing a next-hop node, which we call a forwarding node, when node *s* sends a data packet destined for node *d*.

1. Node *s* checks the highest tier (N) in which nodes *s* and *d* belong to different modules. Then, node *s* sets the initial destination module as the *N*-th-tier module to which node *d* belongs.
2. Descending from tier *N*, node *s* chooses a forwarding module in each tier as follows.
  (a) Node *s* chooses a neighboring *N*-th-tier forwarding module ($M_{f}^{N}$) toward the *N*-th-tier destination module according to the path weight.
  (b) Node *s* chooses an (N−1)-th-tier module ($M_{a}^{N-1}$) that belongs to the same module as node *s* in the *N*-th tier and is connected to an (N−1)-th-tier module ($M_{b}^{N-1}$) belonging to $M_{f}^{N}$.
  (c) Node *s* sets the destination module to $M_{a}^{N-1}$ when node *s* does not belong to $M_{a}^{N-1}$. Otherwise, node *s* sets the destination module to $M_{b}^{N-1}$, which is connected to $M_{a}^{N-1}$ and belongs to $M_{f}^{N}$.
3. By iterating Process 2 until *N* becomes zero, node *s* can find a forwarding node.

In this example, node *s* and node *d* belong to the module, $M_{A}^{3}$, in the third tier. Therefore, node *s* chooses a forwarding module from the second tier. At first, because module $M_{A}^{2}$ and $M_{B}^{2}$ are connected directly in the second tier, a forwarding module in the second tier is $M_{B}^{2}$. The first-tier modules at the end of the second-tier virtual link ($M_{A}^{2}$,$M_{B}^{2}$) are assigned to $M_{B}^{1}$ and $M_{E}^{1}$. Therefore, the data packet needs to go through module $M_{B}^{1}$. Then, node *s* needs to choose a first-tier forwarding module to deliver the data packet to module $M_{B}^{1}$. There are two candidates for the path to module $M_{B}^{1}$, and node *s* selects one of them according to path weight. When node *s* chooses module $M_{D}^{1}$ as the first-tier forwarding module, the data packet needs to go through node *e* because the end nodes of the first-tier virtual link ($M_{C}^{1}$,$M_{D}^{1}$) are assigned to nodes *e* and *i*, respectively. Finally, node *s* chooses node *a* as a forwarding node and sends the data packet to it to deliver the data packet to node *e*. By the same procedure, each node that receives a data packet chooses a forwarding node.

### 5.2. Routing Tables

In this section, we describe the tables that need to be managed by each sensor node for forwarding the data packets. To realize the routing algorithm mentioned in Section 5.1, three types of tables are needed. One is a routing table used to decide a forwarding module from neighbors in each tier; another is a connection table used to manage the (N−1)-th-tier module identifiers, which are assigned to the endpoint modules of *N*-th-tier virtual links; the last is a long-link table used to choose a forwarding node to be assigned to a path for a virtual long-distance link. Each node requires *N* routing tables, (N−1) connection tables and at most one long-link table in order to determine a forwarding node, where *N* is the number of tiers to which the node belongs. Nodes not belonging to any physical paths that are part of a virtual long-distance link do not need a long-link table.

The *N*-th-tier routing table is constructed for routing between (N−1)-th-tier modules belonging to the same *N*-th-tier module. When node *n* belongs to the *N*-th-tier virtual module $M_{A}^{N}$, it records entries of all of the (N−1)-th-tier modules belonging to $M_{A}^{N}$ in its *N*-th-tier routing table. As a particular consequence, this permits routing between any pair of nodes belonging to the same first-tier module by using the zeroth-tier routing table.

The *N*-th-tier connection table converts *N*-th-tier forwarding module identifiers to the (N−1)-th-tier module identifiers associated with the (N−1)-th-tier module belonging to the *N*-th-tier forwarding module. Thus, when we talk about a “connected module” of the virtual module $M_{A}^{N}$, we mean an (N−1)-th-tier virtual module that belongs to the *N*-th-tier virtual module $M_{A}^{N}$ and is connected via an (N−1)-th-tier virtual link to an (N−1)-th-tier virtual module belonging to a different *N*-th-tier virtual module. Because there may be more than one (N−1)-th-tier connected module pointing toward a single *N*-th-tier virtual module, multiple entries for a single *N*-th-tier virtual module are allowed in the *N*-th-tier connection table.

The long-link table is used for routing between nodes that are connected by a virtual link, but cannot communicate directly with each other due to the distance between them. We assign a physical multi-hop path to this kind of virtual long-distance link. Then, each node along the assigned path holds a long-link table for use in choosing a forwarding node toward the destination end of the virtual long-distance link.

### 5.3. Path Recovery after Node Failures

When a node needs to find an alternative path because of the failure of a neighbor node, it is necessary to separately consider two cases: discovery of the minimum-weight path between two nodes that belong to the same first-tier module and recovery of a virtual long-distance link. Moreover, recovery of a virtual long-distance link can be considered for two different cases, as follows.

1. Failure of the relaying node of a path assigned to an *N*-th-tier virtual long-distance link (0≤N).
2. Failure of the end node of an *N*-th-tier virtual long-distance link (0≤N).

In Situation 2, there is no way of selecting an alternative end node for recovery of the virtual long-distance link. By assigning some redundant nodes as the end node of a virtual link, the virtual link can be recovered. In this paper, however, the node does not recover the virtual long-distance link in this situation, and the virtual long-distance link is lost, because we want to evaluate the adaptivity of the initial virtual topology. Therefore, we describe a method for the discovery of an alternative path in only Situation 1 for the recovery of a virtual long-distance link.

The methods are based on finding a reverse path by a flooding control message. To detect the failure of a neighboring node, each node periodically broadcasts a Hello packet and constructs a table of neighbors. When a node detects the failure of a neighboring node via the absence of that node’s Hello packet, it will flood a control message when it is necessary to find an alternative path. In some cases, nodes in the VWSN need to send many Hello packets. Meanwhile, there is a case that nodes detect the failure of a node only when the end-to-end communication failed. Although managing devices is a crucial viewpoint of network design, our scope is to show the adaptivity of the VWSN topology, and a method of resource management is out of scope of this paper.

We note that the flooding range of control packets can be restricted in a first-tier module for the discovery of the alternative path between two nodes that belong to the same first-tier module. This reduces the number of control packets involved in forwarding. In contrast, in the recovery of a path crossing some modules, an alternative path may not be able to be found without wide-range flooding.

Additionally, we need to consider a method that can discover a route between two *N*-th-tier modules when all of the virtual links between them have been lost. The details of the method for this are shown in Section 5.3.4.

#### 5.3.1. Path Recovery between Two Nodes Belonging to the Same First-Tier Module

Each node periodically broadcasts a Hello packet. When node *n* does not receive a Hello packet from node *i* for a certain time, node *n* considers node *i* to have failed. Then, node *n* needs to find an alternative path for any path whose forwarding node is node *i*.

For this, node *n* floods a route request packet to each destination node whose forwarding node from node *n* is node *i*. After a fixed time $t_{reply}$ since the destination node *d* has received the first route request packet from node *n*, it chooses the route request packet with the shortest path and sends a route reply packet to node *n* along the reverse path contained in that packet. Each node on the reverse path updates its zeroth-tier routing table when it receives the route reply packet. Note that the flooding range of the route request packet can be restricted within the module during this path recovery.

#### 5.3.2. Recovery of a Zeroth-Tier Virtual Long-Distance Link

When recovering a zeroth-tier virtual long-distance link, the flooding range of control packets can be restricted to a first-tier module. Therefore, a zeroth-tier virtual long-distance link can be reassigned to an alternative shortest physical path with low overhead.

When a node assigned as a relay node in a zeroth-tier virtual long-distance link (s,d) detects the failure of the forwarding node along (s,d), it sends an error detection packet to source node *s* of the virtual long-distance link, via the reverse path of (s,d). After node *s* receives the error detection packet, it carries out the same route discovery method mentioned in Section 5.3.1. The difference from that method alone is that the table to be updated is the long-link table.

#### 5.3.3. Recovery of an *N*-th-Tier Virtual Long-Distance Link

For recovery of an *N*-th-tier virtual long-distance link, wide flooding may be necessary because a physical path assigned to an *N*-th-tier virtual long-distance link may traverse many first-tier modules. Therefore, we adopt a patching method for the recovery of an *N*-th-tier virtual long-distance link as a way to reduce overhead. The obtained path may not be the shortest path, because we do not find an end-to-end path; however, the recovery time and the overhead can be reduced by not insisting on the optimal path.

In recovering the *N*-th-tier virtual long-distance link (s,d) assigned to path $\left[s\;\left(=p_{0}\right),p_{1},p_{2},\cdots ,p_{l-2},d\;\left(=p_{l-1}\right)\right]$ from the failure of node $p_{i}\;\left(0<i<l-1\right)$, each node $p_{j}\;\left(0\leq j<i-1\right)$ floods a route request packet and searches a path to each node $p_{k}$ where (i+1≤k≤l−1). Here, the range of flooding is restricted by the time-to-live (TTL) of the route request packets.

#### 5.3.4. Path Recovery between Two *N*-th-Tier Modules

We suppose that a virtual long-distance link is lost when an end node of the virtual long-distance link fails or the virtual long-distance link cannot be recovered within a certain time. When all physical paths assigned to an *N*-th-tier (2≤N) virtual long-distance link are lost, the *N*-th-tier routing tables should be updated to route a data packet in the *N*-th tier. Although the principal idea is the same as that of the method shown in Section 5.3.1, flooding in an *N*-th-tier virtual network is different from general flooding. We call this modified form *N*-th-tier flooding.

*N*-th-tier flooding can be realized by multicasting among only connected nodes, because a connected node list corresponding to an *N*-th-tier module list is required for path recovery between two *N*-th-tier modules. When the connected node tries to find an alternative route between *N*-th-tier modules, it sends an upper route request packet to the other connected nodes belonging to its own first-tier module. Then, that node and each of the connected nodes that received an upper route request packet sends the upper route request packet to all connected nodes that belong to neighboring first-tier modules. When a connected node belonging to the destination module receives an upper route request packet, it sends an upper route reply packet to the sender of the upper route request.

## 6. Simulation Evaluation of the Dynamic VWSN

In our previous work [13], we showed how we connect the modules to construct a VWSN network having robust connectivity and a robust path length against nodes’ failure. However, the robust connectivity does not guarantee the reachability of the network. Because the congestion or burst traffics can lead to the loss or inconsistency of table information, robust connectivity is not enough for reliable communication on the VWSN network. Therefore, we show the adaptivity involved in the VWSN network and how to connect modules to construct a reliable VWSN network.

We call our proposed method the brain-inspired configuring method (BICM). Because the BICM method characterizes nine distinct configuration patterns, according to the choice of a combination of intra and inter, we identify the specific pattern by BICM(intra,inter). In consideration of the results shown in [13], we evaluate the VWSN topology constructed by BICM(ll,LL), which is one of the most robust ones against node removal. For comparison, we also evaluate the VWSN topologies constructed by BICM(hh,HH) and by the bio-inspired small-world network construction method (the bio-inspired method) [29]. In our previous work, we showed that the VWSN topology constructed by BICM(hh,HH) is one of the most vulnerable to node removal in the targeted attack mode of the nine strategies. Therefore, we expect that the adaptivity of the VWSN topology of BICM(hh,HH) is also low. We describe the bio-inspired method briefly in Section 6.1.

### 6.1. Bio-Inspired Techniques for Achieving Small-World Properties

The target of the bio-inspired method is WSNs with non-uniform node density. To achieve small-world properties in such WSNs, the method uses bio-inspired techniques [29]. We regard the constructed topology as a virtual topology, although this method is not for constructing a virtual topology. The bio-inspired method consists of two steps: clustering by using a lateral inhibition technique and identifying nodes for constructing long-distance links by using a flocking technique. After the clustering process, all nodes are associated with the maximum-degree cluster head within *η* hops, where *η* restricts the maximum hop distance of the cluster. In the bio-inspired method, a long-distance link is embedded between a peripheral and a centroid node of a cluster for the efficient reduction of average path length. A centroid node is a node with the maximum closeness centrality among nodes in the cluster, and a peripheral node is a node that is located at the boundary of the cluster. Each peripheral node randomly selects the beam length, subject to the restriction by the maximum antenna elements Φ. Then, it looks for centroid nodes within range of a beam of the selected length and nominates potential endpoints of a long-distance link. Each centroid node that is already connected to a neighboring peripheral node is excluded from the set of candidates. Finally, a long-distance link is constructed to the candidate centroid node possessing the highest minimum hop count from the peripheral node.

### 6.2. Evaluation Metrics

In this paper, we define that reliability of a VWSN topology consisting of its robustness and adaptivity. The robustness of a VWSN topology, which is defined as the ability to keep its connectivity high and path length short even when nodes are removed, is shown in our previous work [13]. Here, therefore, we evaluate the adaptivity of a VWSN topology. In this paper, we define adaptivity as the ability to quickly find an alternative path after node failure. To evaluate the adaptivity of the VWSN topology, we evaluate the time ($T_{recovery}$) needed for the data delivery ratio to recover to at least 99.9% after the first sensor node fails. We also evaluate the number of control packets that were sent to find an alternative path ($CP_{recovery}$) after node failure. We evaluate the data delivery ratio from t−500 (in s) to *t*, where *t* is an arbitrary time in the simulation. As we did in our previous paper, we assume two removal modes: random failure and targeted attack. The node to be removed at the next time step is selected randomly in random failure mode, and the node with the highest degree is selected in targeted attack mode.

### 6.3. Evaluation Environments

We evaluate the adaptivity of a VWSN topology constructed from the network, which is composed of two sensor networks and one wired link. Each of the sensor networks consists of 150 sensor nodes deployed in a domain of size $1000\times 1000\;m^{2}$. For one of the sensor networks, we deploy 150 sensor nodes at randomly-selected positions in the rectangular area with corners, denoted in meters along the coordinate axes of the domain, at (0,0) and (400,1000); we deploy the other 150 sensor nodes at randomly-selected positions in the rectangular area given by (600,0) and (1000,1000). Additionally, one wired link connects the two sensor networks, and its endpoint nodes are static once they are chosen. We assume the wireless communication range is 100 m.

In this simulation, we construct a three-tiered VWSN topology. We use OMNeT++ [30] to perform the simulation experiments, and the parameter settings are shown in Table 1. When the physical topology described above is used, the value of $E^{M_{x}^{1}}+E^{M_{y}^{1}}$ is comparatively high, which means that many virtual links are added between first-tier modules; this results in low modularity. To correct for this, we set $C_{\mathrm{inter}}^{1}$ to a value lower than $C_{\mathrm{inter}}^{N}$ in the higher tier.

The flow of simulation is described below.

1. The VWSN provider receives, from a user, a request to construct a new VWSN topology and data on traffic patterns.
2. The VWSN provider constructs a VWSN topology and calculates the routing tables, connection tables and long-link tables for each node.
3. The VWSN provider informs the gateway about the tables of each node and the traffic pattern.
4. The gateway sends the information to each node.
5. Each node sends a data packet periodically according to the received traffic pattern.

The traffic pattern consists of some traffic flow information: a source node, a destination node and a flow rate. In this paper, flow rates are randomly selected from among $\frac{1}{10}$, $\frac{1}{20}$, $\frac{1}{30}$, $\frac{1}{40}$, $\frac{1}{50}$, $\frac{1}{60}$, $\frac{1}{70}$, $\frac{1}{80}$, $\frac{1}{90}$ and $\frac{1}{100}$. The number of flows is 0.2% of the number of all of the possible combinations of two nodes included in the VWSN topology. The pairs of source node and destination node are also selected randomly. The TTL of each data packet is set to 50.

**Figure 4:**
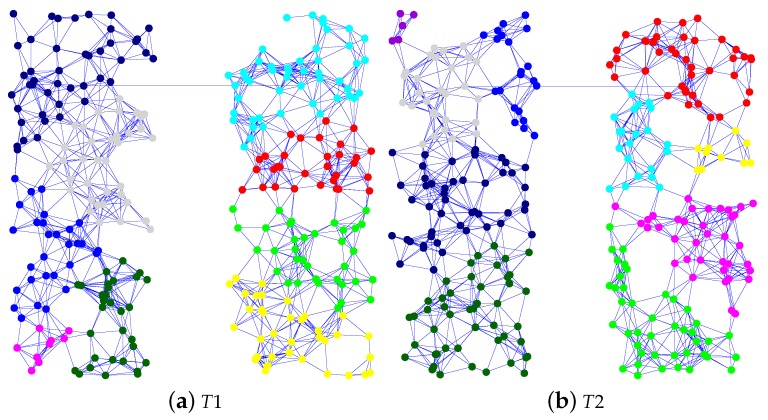
Physical topology and the result of modular division by the Newman algorithm.

From the simulations, we identified the reasons for failure to recover the data delivery ratio. These are listed in Table 2. Physical topology and the result of modular division have a strong influence on adaptivity within our proposed method and can lead to different reasons for failure to recover the data delivery ratio. Therefore, we use five physical topologies and perform 100 trials with each topology. In this paper, we show the results of only two physical topologies; the results for the omitted topologies show the same characteristics. We call these physical topologies T1 and T2. Each physical topology with the result of modular division by the Newman algorithm is shown in Figure 4. Each color shows the group of each module.

### 6.4. Adaptivity of the VWSN Topology against Random Failure

In this section, we evaluate the adaptivity of the VWSN topology against random failure. The simulation time is 10,000 s; after 5000 s have elapsed, one node fails every 10 s until 30 nodes have failed. We use one hundred patterns of node failure, each corresponding to a trial. In each pattern, 30 nodes are randomly selected to fail, and the order of failure is random.

To compare the adaptivity of a physical topology with that of a VWSN topology, we try to evaluate the adaptivity of a physical topology in which we apply shortest-path routing to the topology. However, the simulation cannot be finished. Because all nodes have routing table entries to all other nodes, several nodes generate more than one hundred route request packets after failure of a single node. This leads to frequent packet collision and high packet loss. When a Hello packet is lost, neighboring nodes erroneously detect the failure of the sending node and generate many unnecessary route request packets. From this result, the division of a physical topology into small sub-topologies is an effective method for avoiding this type of problem.

Table 3 shows the rate of recovering the data delivery ratio in each combination of a physical topology and a method of constructing a VWSN topology. The recovery rate is the ratio between the number of trials in which all of the flows can reach the destination node and the total number of trials. In the simulation, phy indicates the physical topology. We use $R_{recovery}$ to denote the rate of recovering the data delivery ratio.

Table 3 shows that $R_{recovery}$ for the bio-inspired topology is zero. There are some connected centroid nodes with a high degree in the virtual topology constructed by the bio-inspired method. The failure of a few of these is fatal because they are essential for connectivity. Note that the routing algorithm mentioned in Section 5 restricts the communication between modules because we keep nodes’ overheads low by reducing their managing information on tables. A data packet must pass through the connected node of the module if the destination node of the data packet belongs to the different module. This means that, in the bio-inspired method, the virtual link connecting different modules is ignored when an endpoint node is neither a centroid node nor a peripheral node. Therefore, a VWSN topology constructed by the bio-inspired method seems to fragment easily into subnetworks because of the routing algorithm, even though it is highly robust on connectivity against random failure, as discussed in our previous work [13]. Moreover, these topologies generate many control packets as part of the route recovery mechanism in upper tiers. This results in congestion and the loss of control packets.

In the topologies created according to our proposal, the physical topology determines whether $R_{recovery}$ is high or low. As an example, when there are some sparse areas in the physical topology, it is difficult to find an alternative path by flooding because of collisions among control packets or isolation in the physical or virtual topology. When we compare values of $R_{recovery}$ among different physical topologies, the differences are small between methods.

**Figure 5:**
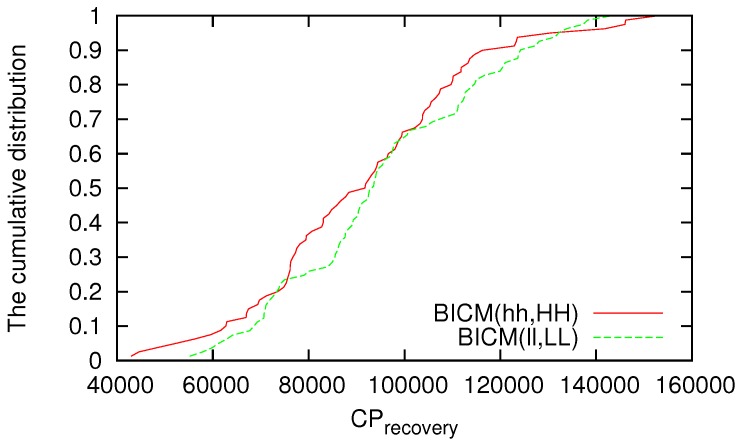
Cumulative distribution of the number of control packets $\left(CP_{recovery}\right)$ when nodes are removed by random failure.

**Figure 6:**
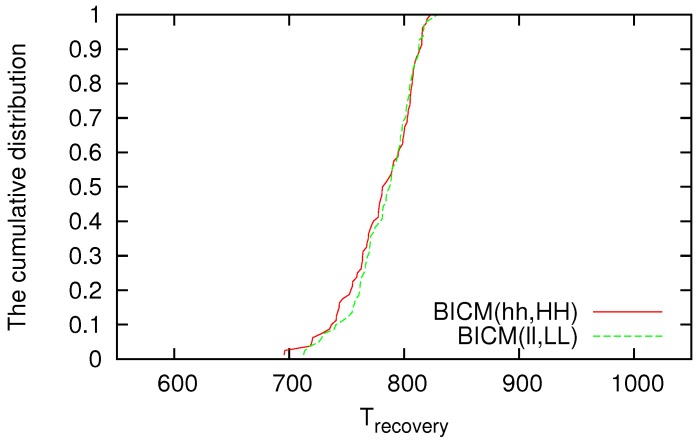
Cumulative distribution of the time needed for the data delivery ratio to recover to over 99.9% $\left(T_{recovery}\right)$ when nodes are removed by random failure.

We investigate the cumulative distributions of $CP_{recovery}$ and $T_{recovery}$ against random failure; however, few differences can be seen among the strategies for inter in each topology. Examples of the cumulative distributions of $CP_{recovery}$ and $T_{recovery}$ are shown in Figure 5 and Figure 6, respectively. In Figure 5, $CP_{recovery}$ is mapped on the horizontal axis, and the cumulative number of trials in which the data delivery ratio recovers before $CP_{recovery}$ has elapsed is mapped on the vertical axis. Similarly, in Figure 6, the horizontal axis reflects $T_{recovery}$, and the vertical axis reflects the cumulative distribution of the number of trials in which the data delivery ratio recovers before $T_{recovery}$ has elapsed. Although the difference according to the method is small for the cumulative distribution of $CP_{recovery}$ or $T_{recovery}$ against random failure, when a VWSN is constructed by BICM(hh,HH), there are some trials whose $CP_{recovery}$ and $T_{recovery}$ are smaller (i.e., better) than those for topologies constructed by other methods. This is because when a node with a high degree is selected as a connected node in BICM(hh,HH), the endpoints of virtual links between modules tend to be concentrated to a small number of nodes. Therefore, the probability that a connected node fails in random failure is low, allowing the recovery of the zeroth-tier routing table unless a connected node fails.

The number of trials in which either the virtual or physical topology fragments is shown in Table 4, and the number of trials in which the data delivery ratio does not recover because of the inconsistency in tables is shown in Table 5. In this, phy identifies the physical topology. Blank entries in Table 4 reflect that the VWSN topology constructed by the bio-inspired method does not have tiers higher than the first tier.

The values of phyFrag are the same in each physical topology, because we use the same node failure patterns. The value of L0Frag for BICM(hh,HH) and BICM(ll,LL) is the same because the results of modular division by the Newman algorithm are almost identical between the cases. Whether the value of L1Frag is high or low depends on the physical topology. For topologies constructed according to our proposed method, it is easy to fragment the first-tier virtual topology into physical topology T2. There are two main reasons for this. One is that there is a first-tier module with a small number of nodes, and the other is that there is a first-tier module whose degree in the first tier is one. The point in common between these is that some first-tier modules contain few connected nodes. Therefore, the failure of a few connected nodes in a first-tier module that either has few nodes or has degree one in the first tier results in the fragmentation of the module. When we use the bio-inspired method, few connected nodes exist in their respective first-tier modules, and each module is small. Therefore, fragmentation of the topology is more likely than with our proposed method.

In Table 5, T1UF has the highest value for each method. This is because it is comparatively difficult to find an alternative route in the upper tier. Particularly when using the bio-inspired method, T1UF is nearly the same as the number of trials. This near parity results from the congestion and loss of control packets around the connected nodes, which attract a large number of links.

From the above, the adaptivity of VWSN topologies constructed by our proposal is not markedly different. However, in a few trials, $T_{recovery}$ is notably smaller in the topology constructed by BICM(hh,HH). This suggests that the adaptivity to random failure of a VWSN topology constructed by BICM(hh,HH) is comparatively high.

### 6.5. Adaptivity of the VWSN Topology against Targeted Attacks

In this section, we evaluate the adaptivity of the VWSN topology against targeted attacks. The simulation time is 10,000 s; after 5000 s have elapsed, one node fails for every 10 s until 30 nodes have failed. Nodes fail in descending order of initial degree in the VWSN topology, without adjusting degrees after each failure and choosing arbitrarily among nodes of equal degree.

The values of $R_{recovery}$, average $T_{recovery}$ and average $CP_{recovery}$ for each method are summarized in Table 6. The number of trials in which the virtual or physical topology becomes fragmented is shown in Table 7, and the number of trials in which the data delivery ratio fails to recover due to the inconsistency in the tables is shown in Table 8. As elsewhere, phy indicates the physical topology. The blanks in Table 6 indicate that the value could not be calculated because the data delivery ratio did not recover.

In physical topology T2, $R_{recovery}$ is quite low when using BICM(hh,HH), but $R_{recovery}$ remains high when using BICM(ll,LL). When using BICM(hh,HH), it is easy to divide a module, and so, the route discovery process must be carried out frequently because high-degree nodes are selected to be connected nodes. This means that many connected nodes fail when nodes are removed by targeted attack. However, the value of $R_{recovery}$ is zero in BICM(ll,LL) for T1. This is because a zeroth-tier topology in a first-tier module becomes fragmented in all trials, as shown in Table 7. In T1, there is a first-tier module whose central area is densely connected. Because the degree of a node in this area is high, this first-tier module becomes fragmented when all of the nodes in the area fail.

From the above, although adaptivity against targeted attack is strongly dependent on physical topology and the method of modular division, the adaptivity of the VWSN constructed by BICM(ll,LL) is the highest in many cases.

Considering all of the results, the physical deployment of sensor nodes and the modular division algorithm is quite important for keeping services running on a VWSN topology. Because sparse areas where link density is low could fragment easily, infrastructure providers should deploy redundant nodes in order to provide stable services. Nodes in dense areas where link density is high can consume more energy than nodes in other areas because they will forward or receive more packets. When a first-tier module has both dense and sparse areas, the energy depletion of nodes in the dense area can result in the fragmentation of the module. To prevent this, infrastructure providers should supply energy to nodes or deploy nodes with high-capacity batteries in areas where many nodes are to be deployed. Alternatively, VWSN providers should construct a first-tier module in which the link density is homogeneous.

### 6.6. Discussion of Memory Utilization

In this section, we estimate the amount of memory needed to store the tables used for the routing algorithm shown in this paper. For the *N*-th-tier routing table, the number of entries that each node needs to hold is the sum of the number of *N*-th-tier modules that belong to the (N+1)-th-tier module of that node (excluding the *N*-th-tier module of the node) and the number of neighboring *N*-th-tier modules that belong to a different (N+1)-th-tier module. Therefore, the number of entries $Z_{RT}^{N}\left(n\right)$ of the *N*-th-tier routing table when node *n* belongs to an *N*-th-tier module $M_{a}^{N}$ and an (N+1)-th-tier module $M_{x}^{N+1}$ is defined as follows:

(4) $$Z_{RT}^{N}\left(n\right)=|Sub^{N}\left(M_{x}^{N+1}\right)\left|-1+\right|N^{N}\left(M_{a}^{N}\right)\backslash Sub^{N}\left(M_{x}^{N+1}\right)|$$

Here, $n\in M_{a}^{N}$ and $n\in M_{x}^{N+1}$. $Sub^{N}\left(M_{x}^{N+1}\right)$ is the set of *N*-th-tier modules that belong to the (N+1)-th-tier module $M_{x}^{N+1}$, and $|Sub^{N}\left(M_{x}^{N+1}\right)|$ is the cardinality of $Sub^{N}\left(M_{x}^{N+1}\right)$. The second term means that $M_{a}^{N}$ is not included as the destination module in the *N*-th-tier routing table. $N^{N}\left(M_{a}^{N}\right)$ is the set of *N*-th-tier modules that neighbor $M_{a}^{N}$, and $N^{N}\left(M_{a}^{N}\right)\backslash Sub^{N}\left(M_{x}^{N+1}\right)$ is the relative complement of $Sub^{N}\left(M_{x}^{N+1}\right)$ in $N^{N}\left(M_{a}^{N}\right)$. Then, $|N^{N}\left(M_{a}^{N}\right)\backslash Sub^{N}\left(M_{x}^{N+1}\right)|$ is the number of *N*-th-tier modules that are connected to $M_{a}^{N}$ and belong to an (N+1)-th-tier module other than $M_{x}^{N+1}$. From the above, the total number of entries $Z_{RT}\left(n\right)$ of the routing tables held by node *n* is

(5) $$Z_{RT}\left(n\right)=\sum_{i}Z_{RT}^{i}\left(n\right),$$

where *i* is the identity of each tier composing the VWSN topology.

Because the number of zeroth-tier modules, which are nodes belonging to the same first-tier module, is the largest of all of the tiers, the size of the zeroth-tier routing table is dominant in many cases.

For the *N*-th-tier connection table, the number of entries that each node needs to hold is the number of (N−1)-th-tier virtual links that connect an (N−1)-th-tier module belonging to the *N*-th-tier module of the node with another (N−1)-th-tier module belonging to a neighboring *N*-th-tier module. In our proposal, the number of (N−1)-th-tier virtual links added by mapping the *N*-th-tier virtual link depends on the number of (N−1)-th-tier virtual links embedded in the *N*-th-tier VWSN topology. Therefore, the number of entries $Z_{CT}^{N}\left(n\right)$ of an *N*-th-tier connection table for node *n* that belongs to $M_{a}^{N}$ is as follows:

(6) $$Z_{CT}^{N}\left(n\right)=\sum_{M_{y}^{N}\in N^{N}\left(M_{a}^{N}\right)}\left\lceil C_{\mathrm{inter}}^{N}\left(E^{M_{a}^{N}}+E^{M_{y}^{N}}\right)\right\rceil$$

Here, $n\in M_{a}^{N}$. Therefore, the total number of entries $Z_{CT}\left(n\right)$ of the connection tables held by *n* is

(7) $$Z_{CT}\left(n\right)=\sum_{i}Z_{CT}^{i}\left(n\right),$$

where *i* is the identity of each tier composing the VWSN topology.

Although the number of zeroth-tier virtual links is the largest among all of the tiers, we can tune the parameter $C_{\mathrm{inter}}^{N}$ separately for each tier. In this paper, because we set $C_{\mathrm{inter}}^{N}$ to a small value, the size of the connection tables is smaller than that of the routing tables.

For the long-link table, the number of entries that each node needs to hold, denoted by $Z_{LT}\left(n\right)$, is the number of virtual links to which it is assigned as a relay node or an end node. Because the number of entries of a long-link table depends entirely on the specific node, we cannot easily estimate the size of the long-link table. Some nodes will have an empty long-link table; others will have a large number of entries in the long-link table. In our evaluation environment, the two nodes connected by the wired link and the nodes around those end nodes have a large number of entries in their long-link tables, because all traffic between the two sensor networks must go through the wired link. However, the number of entries of the long-link tables is much less than that of the routing tables because of the large number of zeroth-tier modules. Because the sizes of the long-link tables depend on the method of modular division, further investigation into the effect of the choice of method of modular division will be needed.

Then, we derive the expectation of the total number of entries of each *N*-th-tier table, denoted by $\langle Z_{RT}^{N}\rangle$, $\langle Z_{CT}^{N}\rangle$ and $\langle Z_{LT}^{N}\rangle$, respectively. Let us define the expectation of each variable as follows. $\langle |Sub^{N}\left(M^{N+1}\right)|\rangle$, $\langle E^{M^{N}}\rangle$ and $\langle k_{M^{N}}\rangle$ denote the expectation of the number of *N*-th-tier modules belonging to an (N+1)-th-tier module, the expectation of the number of (N−1)-th-tier virtual links in an *N*-th-tier module and the expectation of the degree of an *N*-th-tier module, respectively.

We calculates the expectation of the number of *N*-th-tier modules that are connected to an *N*-th-tier module and belong to an (N+1)-th-tier module other than the (N+1)-th-tier module that the *N*-th-tier module belongs to, denoted by $\langle |N^{N}\left(M_{a}^{N}\right)\backslash Sub^{N}\left(M_{x}^{N+1}\right)|\rangle$. The expectation of the total number of *N*-th-tier virtual links that connected *N*-th-tier modules belonging to an (N+1)-th-tier module and *N*-th-tier modules belonging to other (N+1)-th-tier modules equals $\langle Z_{CT}^{N+1}\rangle$. Then, the probability that the number of *N*-th-tier virtual links connecting an *N*-th-tier module and other *N*-th-tier modules belonging to other (N+1)-th-tier modules is *x*, denoted by *p*, is as follows:

(8) p=〈ZCTN+1〉x1〈|SubN(MN+1)|〉x1−1〈|SubN(MN+1)|〉〈ZCTN+1〉−x

Because of the binomial distribution, the following satisfies:

(9) $$\langle |N^{N}\left(M_{a}^{N}\right)\backslash Sub^{N}\left(M_{x}^{N+1}\right)|\rangle =\frac{\langle Z_{CT}^{N+1}\rangle }{\langle |Sub^{N}\left(M^{N+1}\right)|\rangle }$$

Then, $\langle Z_{RT}^{N}\rangle$ is calculated as follows:

(10) $$\langle Z_{RT}^{N}\rangle =\langle |Sub^{N}\left(M^{N+1}\right)|\rangle -1+\frac{\langle Z_{CT}^{N+1}\rangle }{\langle |Sub^{N}\left(M^{N+1}\right)|\rangle }$$

From Equation (6), $\langle Z_{CT}^{N}\rangle$ is calculated as follows:

(11) $$\langle Z_{CT}^{N}\rangle =\left\lceil 2C_{\mathrm{inter}}^{N}\langle k_{M^{N}}\rangle \langle E^{M^{N}}\rangle \right\rceil$$

As mentioned above, we cannot easily estimate the size of the long-link table. Therefore, we use $\langle Z_{LT}^{N}\rangle$ as a parameter in the following discussion.

Then, we discuss how much memory size is required for the tables. Let us denote $b_{r}^{N}$, $b_{c}^{N}$ and $b_{l}$ as the required size of memory of an entry of *N*-th-tier routing table, *N*-th-tier connection table and long-link table in bytes, respectively. Then, the required total memory size for tables of node *n*, denoted by B(n), is

(12) $$B\left(n\right)=\sum_{i}\left(b_{r}^{i}Z_{RT}^{i}\left(n\right)+b_{c}^{i}Z_{CT}^{i}\left(n\right)\right)+b_{l}Z_{LT}\left(n\right).$$

The entries in the routing table in the zeroth tier consist of a destination node, a forwarding node for the destination node and path weight. The entries in the routing table for the higher tier consist of an identifier of a destination module, a forwarding module for the destination module and path weight. Because of the difference of the scale of the number of nodes/modules in each tier, the memory size for the identifier of modules can be smaller in a higher tier. Therefore, we assume that the memory size of the identifier for nodes and modules are two bytes and one byte, respectively, here. We treat the path weight as the float type variable (four bytes). Then, $b_{r}^{0}=8$ and $b_{r}^{N}=6$ where N>0.

When node *n* belongs to the *N*-th-tier virtual module $M_{A}^{N}$, it holds an *N*-th-tier connection table whose entries consist of *N*-th-tier neighboring module identifiers ($M_{B}^{N}$), the module identifier of (N−1)-th-tier connected modules ($M_{X}^{N-1}$) belonging to $M_{A}^{N}$ and the module identifiers of (N−1)-th-tier connected modules ($M_{Y}^{N-1}$) belonging to $M_{B}^{N}$. Because a zeroth-tier module denotes a node, $b_{c}^{1}=6$ and $b_{c}^{N}=4$ where N>1.

The entries of a long-link table consist of the end nodes of a virtual long-distance link, the next and previous forwarding nodes and a hop count from the source node of the virtual long-distance link. Because we set TTL to 50 in our evaluation, one byte is enough for a hop count from the source node of the virtual long-distance link. Then, $b_{l}=9$.

From the above assumptions, Equation (12) can be rewritten as

(13) $$B\left(n\right)=8Z_{RT}^{0}\left(n\right)+\sum_{i>0}\left(6Z_{RT}^{i}\left(n\right)\right)+6Z_{CT}^{1}\left(n\right)+\sum_{i>1}\left(4Z_{CT}^{i}\left(n\right)\right)+9Z_{LT}\left(n\right).$$

Now, we assume a two-tiered structure for one application and a situation in which $\langle |Sub^{0}\left(M^{1}\right)|\rangle =100$, $\langle |Sub^{0}\left(M^{2}\right)|\rangle =10$, $\langle E^{M^{1}}\rangle =2000$, $\langle k_{M^{1}}\rangle =5$ and $\langle Z_{LT}\left(n\right)\rangle =10$. Because the expected number of nodes in a first tier module is 100 and the expected number of total first-tier modules is 10, the total number of nodes in the VWSN network is 1000. From Equations (10) and (11), the expectation of the required total memory size for tables of a node is 2152 bytes.

Next, we assume a three-tiered structure for one application and a situation in which $\langle |Sub^{0}\left(M^{1}\right)|\rangle =100$, $\langle |Sub^{1}\left(M^{2}\right)|\rangle =10$, $\langle |Sub^{2}\left(M^{3}\right)|\rangle =3$, $\langle E^{M^{1}}\rangle =2000$, $\langle E^{M^{2}}\rangle =20$, $\langle k_{M^{1}}\rangle =5$, $\langle k_{M^{2}}\rangle =2$ and $\langle Z_{LT}\left(n\right)\rangle =20$. In this case, the total number of nodes in the VWSN network is 3000. Then, the expectation of the required total memory size for tables of a node is 2290.8 bytes.

Then, we assume a four-tiered structure for one application and a situation in which $\langle |Sub^{0}\left(M^{1}\right)|\rangle =100$, $\langle |Sub^{1}\left(M^{2}\right)|\rangle =10$, $\langle |Sub^{2}\left(M^{3}\right)|\rangle =3$, $\langle |Sub^{3}\left(M^{4}\right)|\rangle =3$, $\langle E^{M^{1}}\rangle =2000$, $\langle E^{M^{2}}\rangle =20$, $\langle E^{M^{3}}\rangle =3$, $\langle k_{M^{1}}\rangle =5$, $\langle k_{M^{2}}\rangle =2$, $\langle k_{M^{3}}\rangle =3$ and $\langle Z_{LT}\left(n\right)\rangle =30$. In this case, the total number of nodes in the VWSN network is 9000. Then, the expectation of the required total memory size for the tables of a node is 2404.8 byte. Therefore, there is a case that 3 KB of memory on average per node is enough for the routing algorithm shown in this paper even when the four-tiered VWSN is provided.

Note that tables for the routing can be reused by multiple applications. When a user wants to run an application over the integrated VWSN, which comprises multiple VWSNs that may have been deployed for other applications, the same tables for routing in the lower tiers can be used in the integrated VWSN. Additional entries in the tables for routing are needed for the highest tier only. Therefore, the modular structure contributes to memory efficiency in such situations.

### 6.7. Discussion of Approaching Our Considered World from the Current Real World

In this paper, we show an overall architecture that is suitable for constructing and running VWSN services within a VWSN topology for the IoT environment. However, it is often considered that nodes in the WSNs are involved in severe restriction on their processing, energy, memory and storage. In the virtualization scenario, this restriction is more critical because multiple applications, such as in-network processing defined by users or the manager of concurrency, run on the same entity.

The things to process are concurrency management, protocol translation, sensing, actuation, packet processing, signal processing, timer management, routing, route recovery, neighbor nodes’ management and table management. Because they can add a big overhead to nodes, how to manage device resources is a crucial viewpoint of network design. In completely centralized management systems, an overhead for collecting information of nodes explosively grows as the number of nodes in the system increases. However, on the contrary, in decentralized systems, more powerful resources are required for any node in the network.

Khan et al. mentioned that research of the virtualization of WSNs is getting more pertinent because WSNs’ nodes are becoming more powerful [16]. It is expected that this trend will continue, and WSNs’ nodes will get more powerful resources in the future. In our architecture, therefore, sensor nodes process their tasks in a decentralized manner after the VWSN provider deploys the constructed VWSN.

Moreover, some techniques with small overheads can be applied to our architecture. For example, only high-spec connected nodes hold the entire *N*-tier routing tables and are responsible for routing between higher tier modules, while a low-spec node holds only zeroth-tier table and sends its sensing data to only the nearest connected node. Any routing algorithm will do in the zeroth-tier, not only any-to-any routing, but also converge routing to a high-spec node. Many efficient converge routing algorithms for WSNs have been proposed [31]. Here, a method of resource management or an energy-efficient solution is out of the scope of this paper and relies on other research.

Another important aspect is Internet compatibility. It is natural to consider that the VWSN providers or users access the virtualized resources through the Internet. Therefore, we need to address compatibility or connectivity between WSNs and the Internet. As mentioned by many researchers [32], the gateway-based strategy can be one solution to this problem. Our architecture can also gain the compatibility to the Internet by using gateway-based solutions. In our architecture, connected nodes can be seen as gateways between modules or networks. As mentioned in Section 1, we consider that users can select or configure protocols that they use in their applications. Standardized protocols, such as 6LoWPAN, can be also included. Moreover, this idea can be applied to each tier in a VWSN network deployed for an application. As mentioned above, because there are some low-spec nodes in WSNs, an energy-efficient and low overhead routing algorithm can be the first choice in zeroth-tier networks. Then, each connected node, behaving as a gateway, aggregates data packets whose destination is out of the module, encapsulates them for routing in higher tier networks and translates protocols as necessary. In this scenario, although the amount of energy consumption is heterogeneous, the heterogeneity of the nodes’ spec is more general in the future IoT environment because of the existence of multiple vendors and providers. How to manage such heterogeneity is out of our scope, but should be future work.

## 7. Conclusions

In a scenario of the virtualization of WSNs, physical networks can undergo dynamic change, such as the addition or removal of nodes or links or resource assignment to fulfill new user requests. Therefore, reliability is important even when such environmental changes occur. To tackle these problems, we show an overall architecture of constructing and running VWSN services with considering the environmental changes. We define that reliability consists of robustness and adaptivity. In our previous work, we proposed a method for constructing a robust VWSN topology against node failure. In this paper, we conduct a simulation of the practical situation to evaluate the adaptivity of our proposed VWSN topology in consideration of an actual environment.

The results of the simulation experiments showed that the adaptivity of the VWSN topology constructed by BICM(ll,LL) was the highest against target attack, which is consistent with the robustness results. However, when there is a first-tier module whose central area is densely connected, the zeroth-tier virtual topology in the first-tier module becomes fragmented quite easily. To address this problem, it is necessary to more deeply consider the method of modular division and the modular structure. We intend to do so in future work.

We also discussed the memory needed for the tables used in our proposed routing. In our evaluation environment, the size of a zeroth-tier routing table is the most dominant. However, the number of entries of each table depends on the method of modular division, the number of connected modules and nodes and the number of virtual long-distance links. Therefore, further investigation into the relation between the chosen method of modular division and memory efficiency will be needed.

In this paper, we analyzed the properties of a virtual topology composed of only sensor nodes. Therefore, we need to consider the following in future work. First, the method of realizing a virtual link in a physical network should be investigated; packets forwarded along a virtual long-distance link should be conveyed with only a short delay. As candidate methods for this, we intend to consider creating directional beams, increasing omnidirectional transmission range and multi-hop forwarding with variable priority. Second, because there may be multiple demands for constructing VWSNs that compete for resources, such as energy, memory and bandwidth, it is necessary to consider a method that can construct resource-efficient VWSN topologies. Third, we hope to create a protocol for evolving the VWSN topology in response to environmental changes, such as changes in traffic patterns. Due to the modular structure, a small adjustment of a few virtual links between modules should be sufficient to achieve that.

## Figures and Tables

**Figure A1:**
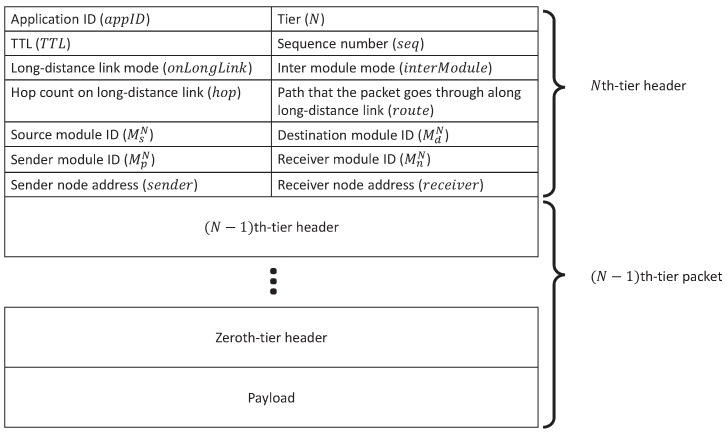
Format of the *N*-th-tier header prepended to (N−1)-th-tier packets.

**Figure A2:**
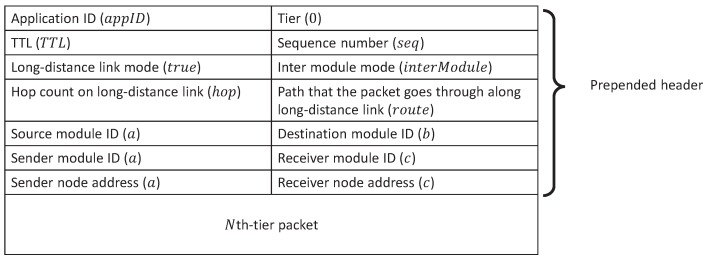
Packet format when the data packet exists on a virtual long-distance link.

**Table 1:** Parameter settings. BICM, brain-inspired configuring method.

Method	Parameter	Value
BICM	$C_{\mathrm{intra}}^{N}$	0.1
	$C_{\mathrm{inter}}^{N}\left(N\neq 1\right)$	0.1
	$C_{\mathrm{inter}}^{1}$	0.01
Bio-inspired	*η*	4
	Φ	6

**Table 2:** List of reasons for the failure to recover the data delivery ratio.

Abbreviation	Description of Reasons for the Recovery Failure
phyFrag	The physical topology fragments into some subnetworks. This leads to unreachable nodes from a source node because no physical path exists between them.
L0Frag	A zeroth-tier virtual topology in the first-tier module fragments into some zeroth-tier virtual subnetworks. This leads to mutually unreachable pairs that belong to the same first-tier module, even when a physical path between them exists.
L1Frag	A first-tier virtual topology in the second-tier module fragments into some first-tier virtual subnetworks. This leads to unreachable pairs of a source first-tier module and a destination first-tier module that belong to the same second-tier module, even when a physical path between them exists.
L2Frag	A second-tier virtual topology in the third-tier module fragments into some second-tier virtual subnetworks. This leads to mutually unreachable pairs of second-tier modules that belong to the same third-tier module, even when a physical path between them exists.
T0UF	A node cannot find an alternative path to a node that belongs to the same first-tier module due to packet loss, when the zeroth-tier virtual topology in the first-tier module is not fragmented.
T1UF	A connected node that belongs to one first-tier module cannot find an alternative path to another first-tier module due to packet loss, when the first-tier virtual topology in the second-tier module is not fragmented.
L0Loop	A loop exists within the first-tier module due to inconsistent weights in the zeroth-tier routing table.
L1Loop	A loop exists within the second-tier module due to inconsistent weights in the first-tier routing table.
LLLost	A data packet cannot arrive at the end node of a virtual long link due to inconsistency in the long-link table.
TTL	A data packet expires, even when an alternative path is found.
$ConT^{N}Up$	A node cannot update its *N*-th-tier connection module table due to packet loss. Then, the node continues to send data packets to a module that is no longer a connected module.

**Table 3:** Rate of recovering the data delivery ratio ($R_{recovery}$) against random failure.

	phy	$R_{\mathrm{recovery}}$
BICM(hh,HH)	T1	0.80
BICM(ll,LL)	T1	0.81
Bio-inspired	T1	0.00
BICM(hh,HH)	T2	0.69
BICM(ll,LL)	T2	0.67
Bio-inspired	T2	0.00

**Table 4:** Number of trials in which the virtual or physical topology fragments when nodes are removed by random failure.

	phy	phyFrag	L0Frag	L1Frag	L2Frag
BICM(hh,HH)	T1	19	17	1	0
BICM(ll,LL)	T1	19	17	0	0
Bio-inspired	T1	19	46	26	
BICM(hh,HH)	T2	6	18	5	0
BICM(ll,LL)	T2	6	18	5	0
Bio-inspired	T2	6	62	63	

**Table 5:** Number of trials in which the data delivery ratio fails to recover due to the inconsistency in tables when nodes are removed by random failure.

	phy	T0UF	T1UF	L0Loop	L1Loop	LLLost	TTL	${\mathrm{ConT}}^{N}\mathrm{Up}$
BICM(hh,HH)	T1	0	5	2	0	0	0	2
BICM(ll,LL)	T1	0	4	3	0	0	0	2
Bio-inspired	T1	11	99	2	0	0	0	2
BICM(hh,HH)	T2	0	14	0	0	0	0	1
BICM(ll,LL)	T2	3	13	1	0	0	0	2
Bio-inspired	T2	10	98	0	0	2	2	7

**Table 6:** The rate of recovering the data delivery ratio $\left(R_{recovery}\right)$, the average time needed for data delivery ratio to recover to over 99.9% $\left(T_{recovery}\right)$ and the average number of control packets $\left(CP_{recovery}\right)$ when nodes are removed by targeted attack.

	phy	$R_{\mathrm{recovery}}$	Average $T_{\mathrm{recovery}}$	Average ${\mathrm{CP}}_{\mathrm{recovery}}$
BICM(hh,HH)	T1	0.16	793.43	$16.66\times 10^{4}$
BICM(ll,LL)	T1	0.00		
Bio-inspired	T1	0.00		
BICM(hh,HH)	T2	0.07	791.87	$24.13\times 10^{4}$
BICM(ll,LL)	T2	0.83	797.07	$18.17\times 10^{4}$
Bio-inspired	T2	0.00		

**Table 7:** Number of trials in which the virtual or physical topology becomes fragmented when nodes are removed by targeted attack.

	phy	phyFrag	L0Frag	L1Frag	L2Frag
BICM(hh,HH)	T1	0	35	48	26
BICM(ll,LL)	T1	0	100	0	0
Bio-inspired	T1	0	88	86	
BICM(hh,HH)	T2	1	7	61	9
BICM(ll,LL)	T2	0	8	0	0
Bio-inspired	T2	0	100	99	

**Table 8:** Number of trials in which the data delivery ratio fails to recover due to the inconsistency in tables when nodes are removed by targeted attack.

	phy	T0UF	T1UF	L0Loop	L1Loop	LLLost	TTL	${\mathrm{ConT}}^{N}\mathrm{Up}$
BICM(hh,HH)	T1	0	49	1	1	0	2	2
BICM(ll,LL)	T1	0	2	4	0	1	0	1
Bio-inspired	T1	7	100	2	0	2	0	11
BICM(hh,HH)	T2	3	57	6	0	0	1	10
BICM(ll,LL)	T2	7	1	2	0	0	1	1
Bio-inspired	T2	8	100	5	0	1	1	8

## Appendix A. Packet Format

In this section, we explain the packet format used to realize the routing mentioned in Section 5.1. The data packet needs to include an application identifier, a source node address, an identifier of the *i*-th-tier module to which the source node belongs (1≤i≤N), a destination node address, an identifier of the *i*-th-tier module to which the destination node belongs and the sender and receiver node addresses of the data packet. These pieces of information are included in the packet by encapsulating a packet recursively and adding the identifiers for the source and destination module in each tier. The packet format is shown in Figure A1. The source node encapsulates the data packet. When the *N*-th-tier packet reaches the destination module in the *N*-th tier, it is unpacked, and the (N−1)-th-tier packet is then routed.

The field “long-distance link mode” is a flag that shows whether the data packet exists on the path assigned to a virtual long-distance link and is routed to the end node of the virtual long-distance link. The field “inter module mode” is a flag that shows whether the data packet exists on the path assigned to a virtual link connecting modules in the first tier or higher. The field “hop count on long-distance link” is the hop count to the source node of a virtual long-distance link, and the field containing the “path on long-distance link” is a list of node addresses for forwarding this packet along the virtual long-distance link. These fields are used for the maintenance of long-link tables.

If the data packet reaches the source node *a* of a virtual long-distance link and node *a* decides to forward it to destination node *b* of the virtual long-distance link, then node *a* encapsulates the packet to indicate that the packet is in long-distance link mode. Then, node *a* queries its long-link table and determines the next-hop node *c* for delivering the packet to node *b* along the virtual long-distance link. The packet format for this situation when the data packet exists on a virtual long-distance link is shown in Figure A2.

## Appendix B. Definition of Weights of Virtual Links and Virtual Modules

In this section, we define the weights of virtual links and virtual modules used in selecting a forwarding module by the method described in Section 5.1. In this paper, the weights of virtual links and virtual modules are calculated from the hop counts of the physical paths.

First, we define the weight of an *N*-th-tier virtual link. Because a virtual module in the zeroth tier is identically a physical node, we define the weight of a zeroth-tier virtual link as the shortest hop count between its end nodes. The definition of the weight of an *N*-th-tier virtual link whose *N*-th-tier endpoint modules are $M_{A}^{N}$ and $M_{B}^{N}$ is

(B1) $$W_{l}^{N}\left(M_{A}^{N},M_{B}^{N}\right)=\frac{1}{|P^{N-1}\left(M_{A}^{N},M_{B}^{N}\right)|}\sum_{\left(x,y\right)\in P^{N-1}\left(M_{A}^{N},M_{B}^{N}\right)}W_{l}^{N-1}\left(x,y\right),$$

where $P^{N-1}\left(M_{A}^{N},M_{B}^{N}\right)$ is the set of (N−1)-th-tier virtual links to which the *N*-th-tier virtual link $\left(M_{A}^{N},M_{B}^{N}\right)$ is assigned; $|P^{N-1}\left(M_{A}^{N},M_{B}^{N}\right)|$ is the cardinality of $P^{N-1}\left(M_{A}^{N},M_{B}^{N}\right)$; and $W_{l}^{N-1}\left(x,y\right)$ is the weight of the (N−1)-th-tier virtual link between module *x* and module *y*. The weight of the *N*-th-tier virtual link between module $M_{A}^{N}$ and module $M_{B}^{N}$ is the mean of the weights of the (N−1)-th-tier virtual links to which it is assigned.

Then, we define the weight of an *N*-th-tier virtual module as the cost of going through the module. We assume that the weight of a virtual module in the zeroth tier is zero. We define the weight of an *N*-th-tier virtual module as the mean of the hop count from one neighboring *N*-th-tier module to another neighboring *N*-th-tier module. This means that the weight of a virtual module changes according to the source and destination of a flow. Therefore, when a flow comes from an *N*-th-tier virtual module $M_{B}^{N}$ and goes to $M_{C}^{N}$, we define the weight of an *N*-th-tier virtual module $M_{A}^{N}$ as

(B2) $$W_{m}^{N}\left(M_{A}^{N},M_{B}^{N},M_{C}^{N}\right)=\frac{1}{|Con\left(M_{B}^{N},M_{A}^{N}\right)|\cdot |Con\left(M_{C}^{N},M_{A}^{N}\right)|}\sum_{\begin{array}{c} x\in Con(M_{B}^{N},M_{A}^{N}),\\ y\in Con(M_{C}^{N},M_{A}^{N}) \end{array}}PW(M_{A}^{N},x,y),$$

where $Con(M_{B}^{N},M_{A}^{N})$ is the set of (N−1)-th-tier connected modules belonging to $M_{B}^{N}$ and connecting to an (N−1)-th-tier connected module of $M_{A}^{N}$, and $\left|Con(M_{B}^{N},M_{A}^{N})\right|$ is the cardinality of $Con(M_{B}^{N},M_{A}^{N})$. Then, $PW(M_{A}^{N},x,y)$ is the weight of the virtual module $M_{A}^{N}$ when a flow comes from an (N−1)-th-tier module *x* and goes to an (N−1)-th-tier module *y*. We define $PW(M_{A}^{N},x,y)$ as

(B3) $$\begin{array}{c} \begin{array}{cc} PW(M_{A}^{N},x,y) & =\min_{p\in Path^{N-1}\left(M_{A}^{N},x,y\right)}(W_{m}^{N-1}\left(M_{p_{1}}^{N-1},x,M_{p_{2}}^{N-1}\right)+\sum_{i=2}^{k-1}(W_{l}^{N-1}\left(M_{p_{i-1}}^{N-1},M_{p_{i}}^{N-1}\right)+\\ & W_{m}^{N-1}\left(M_{p_{i}}^{N-1},M_{p_{i-1}}^{N-1},M_{p_{i+1}}^{N-1}\right))+W_{l}^{N-1}\left(M_{p_{k-1}}^{N-1},M_{p_{k}}^{N-1}\right)\\ & +W_{m}^{N-1}\left(M_{p_{k}}^{N-1},M_{p_{k-1}}^{N-1},y\right)), \end{array} \end{array}$$

where $Path^{N-1}\left(M_{A}^{N},x,y\right)$ is the set of (N−1)-th-tier paths between *x* and *y*. Each element *p* is represented as $\left(M_{p_{1}}^{N-1},\cdots ,M_{p_{k}}^{N-1}\right)$, where elements of $M_{p_{i}}^{N-1}\left(1\leq i\leq k\right)$ belong to $M_{A}^{N}$.

## References

[1] Atzori L., Iera A., Morabito G. (2010). The internet of things: A survey. Comput. Netw..

[2] Islam M.M., Hassan M.M., Lee G.W., Huh E.N. (2012). A survey on virtualization of wireless sensor networks. Sensors.

[3] Ishaq I., Hoebeke J., Moerman I., Demeester P. Internet of things virtual networks: Bringing network virtualization to resource-constrained devices. Proceedings of the IEEE International Conference on Green Computing and Communications (GreenCom 2012).

[4] Khan I., Belqasmi F., Glitho R., Crespi N., Morrow M., Polakos P. (2015). Wireless sensor network virtualization: Early architecture and research perspectives. IEEE Netw..

[5] Wang W., De S., Cassar G., Moessner K. (2015). An experimental study on geospatial indexing for sensor service discovery. Expert Syst. Appl..

[6] Leontiadis I., Efstratiou C., Mascolo C., Crowcroft J. (2012). SenShare: Transforming sensor networks into multi-application sensing infrastructures. Proceedings of the 9th European Conference on Wireless Sensor Networks.

[7] Trakadas P., Leligou H., Zahariadis T., Karkazis P., Sarakis L. (2013). Managing QoS for future internet applications over virtual sensor networks. Future Internet Assembly 2013: Validated Results and New Horizons.

[8] Levis P., Culler D. MatÉ: A tiny virtual machine for sensor networks. Proceedings of the 10th International Conference on Architectural Support for Programming Languages and Operating Systems (ASPLOS-X).

[9] Chu R., Gu L., Liu Y., Li M., Lu X. (2013). SenSmart: Adaptive stack management for multitasking sensor networks. IEEE Trans. Comput..

[10] Fok C.L., Roman G.C., Lu C. (2009). Agilla: A mobile agent middleware for self-adaptive wireless sensor networks. ACM Trans. Auton. Adapt. Syst..

[11] Khan I., Jafrin R., Zahra Errounda F., Glitho R., Crespi N., Morrow M., Polako P. A data annotation architecture for semantic applications in virtualized wireless sensor networks. Proceedings of the 14th IFIP/IEEE Symposium on Integrated Network and Service Management (IM 2015).

[12] Bandara H., Jayasumana A.P., Illangasekare T.H. (2011). A top-down clustering and cluster-tree-based routing scheme for wireless sensor networks. Int. J. Distrib. Sens. Netw..

[13] Toyonaga S., Kominami D., Murata M. Brain-inspired method for constructing a robust virtual wireless sensor network. Proceedings of the 10th International Conference on Computing and Network Communications (CoCoNet 2015).

[14] Papo D., Buldú J.M., Boccaletti S., Bullmore E.T. (2014). Complex network theory and the brain. Philos. Trans. R. Soc. B.

[15] Meunier D., Lambiotte R., Bullmore E.T. (2010). Modular and hierarchically modular organization of brain networks. Front. Neurosci..

[16] Khan I., Belqasmi F., Glitho R., Crespi N., Morrow M., Polakos P. (2015). Wireless sensor network virtualization: A survey. IEEE Commun. Surv. Tutor..

[17] Fischer A., Botero J., Till Beck M., de Meer H., Hesselbach X. (2013). Virtual network embedding: A survey. IEEE Commun. Surv. Tutor..

[18] Rajeshwari P., Shanthini B., Prince M. (2015). Hierarchical energy efficient clustering algorithm for WSN. IDOSI Publ..

[19] Seyed Mahdi Jameii K.F., Dehghan M. (2016). AMOF: Adaptive multi-objective optimization framework for coverage and topology control in heterogeneous wireless sensor networks. Telecommun. Syst..

[20] Qin Z., Denker G., Giannelli C., Bellavista P., Venkatasubramanian N. A software defined networking architecture for the internet-of-things. Proceedings of the 2014 IEEE Network Operations and Management Symposium (NOMS).

[21] Jararweh Y., Al-Ayyoub M., Darabseh A., Benkhelifa E., Vouk M., Rindos A. (2015). SDIoT: A software defined based internet of things framework. J. Ambient Intell. Hum. Comput..

[22] Bullmore E., Sporns O. (2012). The economy of brain network organization. Nat. Rev. Neurosci..

[23] Park C.H., Kim S.Y., Kim Y.H., Kim K. (2008). Comparison of the small-world topology between anatomical and functional connectivity in the human brain. Physica A.

[24] Gallos L.K., Makse H.A., Sigman M. (2012). A small world of weak ties provides optimal global integration of self-similar modules in functional brain networks. Proc. Natl. Acad. Sci. USA.

[25] Achard S., Salvador R., Whitcher B., Suckling J., Bullmore E. (2006). A resilient, low-frequency, small-world human brain functional network with highly connected association cortical hubs. J. Neurosci..

[26] Vertes P.E., Alexander-Bloch A.F., Gogtay N., Giedd J.N., Rapoport J.L., Bullmore E.T. (2012). Simple models of human brain functional networks. Proc. Natl. Acad. Sci. USA.

[27] Samu D., Seth A.K., Nowotny T. (2014). Influence of wiring cost on the large-scale architecture of human cortical connectivity. PLoS Comput. Biol..

[28] Newman M.E. (2006). Modularity and community structure in networks. Proc. Natl. Acad. Sci. USA.

[29] Agarwal R., Banerjee A., Gauthier V., Becker M., Yeo C.K., Lee B.S. (2012). Achieving small-world properties using bio-inspired techniques in wireless networks. Comput. J..

[30] Varga A. (2010). OMNeT++. Modeling and Tools for Network Simulation.

[31] Toyonaga S., Daichi Kominami M.S., Murata M. (2013). Potential-based routing for supporting robust any-to-any communication in wireless sensor networks. EURASIP J. Wirel. Commun. Netw..

[32] Xu K., Qu Y., Yang K. (2016). A tutorial on the internet of things: From a heterogeneous network integration perspective. IEEE Netw..

